# Continuous Non-Invasive Assessment of Segmental Cervical Motion: A Narrative Review and Validation Framework

**DOI:** 10.3390/bioengineering13050584

**Published:** 2026-05-20

**Authors:** Nicole Burtovaja, Sergejs Burtovojs, Yuri Dekhtyar, Ross A. Hauser, Leonids Ribickis

**Affiliations:** 1 Institute of Mechanical Engineering, Aerospace Technologies and Transport, Faculty of Civil and Mechanical Engineering, Riga Technical University, 6B Kipsalas Street, LV-1048 Riga, Latvia; jurijs.dehtjars@rtu.lv; 2 Institute of Industrial Electronics, Electrical Engineering and Energy, Faculty of Computer Science, Information Technology and Energy, Riga Technical University, 12/1 Azenes Street, LV-1048 Riga, Latvia; sergejs.burtovojs@rtu.lv (S.B.); leonids.ribickis@rtu.lv (L.R.); 3 Caring Medical Florida, 9738 Commerce Center Court, Fort Myers, FL 33908, USA; rhprolobook@caringmedical.com

**Keywords:** cervical spine, segmental cervical motion, cervical diagnostics, digital measures, validation framework, wearable sensors, multimodal sensing, dynamic fluoroscopy, telemedicine, longitudinal monitoring

## Abstract

Neck pain is increasingly associated with exposure-dependent dysfunction linked to digitally mediated behaviors, prolonged near-work, sustained postures, and reduced movement variability, whereas cervical assessment remains dominated by static imaging and brief in-clinic examination. This narrative review evaluates why current diagnostic approaches remain poorly suited to the dynamic nature of many contemporary cervical disorders and examines segmental cervical motion as a clinically relevant but insufficiently observed functional target. Evidence from static imaging, dynamic radiographic methods, laboratory motion analysis, wearable inertial sensing, markerless video, and digital measure validation frameworks is synthesized to assess both current capabilities and translational limitations. Dynamic radiographic methods can characterize intervertebral motion with high anatomical specificity, but they are not suitable for scalable longitudinal monitoring. By contrast, wearable and video-based approaches are more compatible with real-world assessment, yet they capture external head–neck kinematics rather than vertebral-level kinematics directly and remain constrained by indirect observability, soft-tissue artifact, and inference uncertainty. On this basis, the review proposes a four-layer framework for continuous non-invasive cervical functional assessment based on sensing, representation, inference, and clinical interpretation, in which segmental cervical behavior is treated as a latent segment-informed functional construct inferred from multimodal external signals and periodically anchored to sparse reference-grade imaging anchors. Segmental motion signatures are consequently positioned as candidate digital measures for longitudinal cervical monitoring, provided that their development is supported by rigorous analytical and clinical validation, explicit uncertainty reporting, and demonstrated incremental clinical value.

## 1. Introduction

Neck pain is one of the most prevalent musculoskeletal disorders worldwide and remains a major contributor to disability across both working-age and older populations. Recent estimates from the Global Burden of Disease Study 2021 indicate that the burden of neck pain remains substantial and is projected to rise further by 2050, largely because of population growth and aging [1]. At the same time, daily exposure patterns have changed markedly. Work and leisure are increasingly shaped by prolonged near-work, sustained sitting, frequent smartphone use, and reduced movement variability, all of which may contribute to symptom aggravation in contemporary neck disorders [2,3,4]. Meta-analytic evidence supports associations between neck pain and smartphone overuse as well as posture-related factors such as forward head posture, reinforcing the clinical relevance of cumulative low-load exposure rather than isolated acute events alone [2,3].

Despite this shift in exposure profile, cervical assessment remains dominated by static imaging and brief in-clinic examination [4]. These approaches remain indispensable for identifying serious structural pathology, including fracture, deformity, neural compression, infection, tumor, and major instability, and they are supported by contemporary imaging guidance for cervical pain and radiculopathy [4,5]. However, they are less well suited to disorders in which symptoms fluctuate with task duration, cumulative loading, or impaired recovery over time. Reviews of diagnostic indicators in neck pain further suggest that robust and consistent clinical markers remain limited across many presentations, especially in non-specific conditions [4,6]. The problem is compounded by persistent clinico-radiographic discordance: degenerative imaging findings are common in asymptomatic individuals, whereas patients with substantial pain and disability may show only modest structural abnormalities [7,8]. Together, these limitations indicate that structure-based assessment alone often provides an incomplete account of everyday cervical dysfunction.

One functional domain that may help narrow this gap is segmental cervical motion, understood as the relative kinematics between adjacent vertebral levels. Unlike global cervical range of motion, segmental cervical behavior may better reflect how movement is distributed across the cervical spine and how load sharing, compensation, and coordination change during sustained postures or repeated tasks. This perspective is consistent with broader evidence that neck disorders frequently involve altered sensorimotor function, including disturbances in postural stability, head–eye control, and joint position sense [9,10]. Dynamic imaging and biomechanical studies further indicate that intervertebral motion characteristics vary across cervical levels, movement directions, age groups, and degenerative states, supporting the view that segmental cervical motion is a distinct and clinically relevant functional domain rather than simply a higher-resolution extension of global range-of-motion testing [11,12,13,14,15].

Yet segmental cervical motion remains difficult to assess in a way that is both anatomically meaningful and scalable for longitudinal use. Dynamic radiographic methods, including quantitative fluoroscopy and dual fluoroscopic imaging systems, can characterize intervertebral motion with high anatomical specificity and useful repeatability under standardized conditions [13,16,17,18]. However, these techniques are episodic, infrastructure-intensive, and poorly suited to continuous monitoring in everyday environments. By contrast, laboratory motion capture, wearable inertial sensing, and markerless video approaches are more compatible with repeated or real-world assessment, but they capture external head–neck kinematics rather than vertebral-level kinematics directly and are therefore constrained by indirect observability, soft-tissue artifact, placement sensitivity, context variability, and uncertainty in inference [19,20,21,22,23,24]. A translational gap therefore persists between reference-grade segmental cervical motion characterization and scalable functional monitoring outside the clinic.

This review addresses that gap from a validation-oriented perspective. Rather than treating segmental cervical motion as a continuously measurable anatomical target in daily life, we argue that segmental cervical behavior is more appropriately conceptualized as a clinically meaningful but indirectly observable latent segment-informed functional construct. On this basis, the review has three aims: first, to examine why current cervical diagnostic pathways remain poorly aligned with time-dependent dysfunction; second, to evaluate segmental cervical motion as a relevant translational target for functional assessment; and third, to outline a framework for continuous non-invasive cervical functional assessment based on multimodal sensing, structured representation, uncertainty-aware inference, and sparse reference-grade imaging anchors. By integrating evidence from cervical diagnostics, motion analysis, scalable sensing, and digital validation science, this review seeks to define a realistic pathway toward longitudinal, functionally grounded cervical assessment.

The contribution of this review is threefold. First, it synthesizes the translational gap between reference-grade segmental cervical motion characterization and scalable real-world monitoring. Second, it proposes a four-layer framework linking sensing, representation, inference, and clinical interpretation for continuous non-invasive cervical functional assessment. Third, it defines segmental motion signatures as candidate digital measures and positions them within a validation pathway based on context of use, BEST terminology, V3/V3+ principles, uncertainty reporting, and falsifiability. To our knowledge, this is among the first reviews to frame this asymmetry as a validation-oriented pathway connecting reference-grade segmental motion characterization with scalable longitudinal monitoring. Thus, the novelty of the manuscript lies not in claiming a validated diagnostic tool, but in defining a structured pathway for developing and validating future cervical digital measures.

## 2. Review Methodology

### 2.1. Review Design

This study was conducted as a validation-oriented narrative review. A narrative design was selected because the literature relevant to continuous non-invasive cervical functional assessment is methodologically heterogeneous and spans multiple domains, including cervical diagnostics, segmental cervical motion characterization, laboratory biomechanics, wearable inertial sensing, markerless motion analysis, candidate digital measures, and validation frameworks in digital health. Under such conditions, a formal systematic review or meta-analysis would be poorly suited to the main objective of the present work, which is not to pool homogeneous effect estimates for a single intervention or outcome, but to synthesize translationally relevant evidence across modalities, levels of measurement, and validation paradigms [25,26,27,28].

### 2.2. Search Strategy and Source Selection

The methodological approach was informed by published guidance on narrative reviews and by quality considerations relevant to non-systematic evidence synthesis [25,27,28]. The search and source-selection process was documented descriptively, while recognizing that the present study was not designed as a systematic or scoping review [26].

The literature search was conducted across three widely used biomedical, engineering, and multidisciplinary databases: PubMed/MEDLINE, Scopus, and Web of Science. Additional targeted searching of reference lists and key methodological papers was performed where necessary. The search covered literature available up to April 2026. Search terms were organized around five thematic domains: (i) neck pain, cervical diagnostics, and time-dependent dysfunction; (ii) segmental or intervertebral cervical motion, including dynamic radiographic methods, quantitative fluoroscopy, and dual fluoroscopic imaging systems; (iii) external head–neck kinematic assessment, including optical motion capture, wearable inertial sensing, and markerless video-based analysis; (iv) digital measures, digital biomarkers, and longitudinal monitoring; and (v) verification, analytical validation, clinical validation, usability, and implementation frameworks relevant to digital health technologies.

Representative search terms included “neck pain”, “cervical diagnostics”, “time-dependent dysfunction”, “segmental cervical motion”, “intervertebral cervical motion”, “dynamic fluoroscopy”, “quantitative fluoroscopy”, “dual fluoroscopic imaging”, “dual fluoroscopic imaging system”, “cervical kinematics”, “optical motion capture”, “wearable sensors”, “inertial measurement unit”, “IMU”, “markerless motion analysis”, “pose estimation”, “digital measures”, “digital biomarkers”, “longitudinal monitoring”, “telemedicine”, “verification”, “analytical validation”, “clinical validation”, “V3 framework”, and “usability validation”. In total, approximately 140 potentially relevant records were identified through database searches, reference-list screening, and targeted searches of key methodological papers. After title-and-abstract relevance screening, 48 sources were selected for inclusion in the final synthesis. These numbers are reported as descriptive screening estimates rather than PRISMA-based counts.

The final set of sources was selected on the basis of conceptual relevance, usefulness for the review aims, and balanced coverage across the five thematic domains. Sources were retained when they contributed directly to the central translational argument of the review, including limitations of current cervical diagnostics, reference-grade segmental cervical motion characterization, scalable external head–neck kinematic assessment, observability constraints and uncertainty in inference, or validation requirements for candidate digital measures. Eligible sources included peer-reviewed original studies, systematic and narrative reviews, methodological papers, consensus-oriented resources, and authoritative framework documents. Conference abstracts, preliminary reports without sufficient detail, and sources not directly contributing to the cervical or digital validation argument were deprioritized. Lumbar-spine studies were considered only when they provided relevant methodological insight for motion analysis or quantitative fluoroscopy and when comparable cervical-specific evidence was limited.

### 2.3. Thematic Synthesis Approach

The included literature was interpreted comparatively rather than exhaustively and was organized into predefined thematic domains aligned with the structure of the review. No formal risk-of-bias assessment or quantitative evidence grading was performed, because the aim was conceptual and translational synthesis rather than pooled evidence evaluation. Particular emphasis was placed on how different evidence streams support a staged translational pathway from reference-grade segmental cervical motion characterization to scalable longitudinal monitoring. The synthesis therefore focused not only on what each modality can measure, but also on what remains unobservable, weakly validated, or difficult to translate into clinically meaningful real-world assessment.

Accordingly, this review was designed not as a comprehensive catalog of all cervical motion technologies, but as a structured synthesis intended to define a realistic functional target for continuous non-invasive cervical functional assessment and to outline a validation-oriented framework for future development.

## 3. Why Current Cervical Diagnostics Remain Misaligned with Time-Dependent Dysfunction

Current cervical diagnostic pathways are designed primarily to identify structural and neurological pathology, including fracture, ligamentous disruption, radiculopathy, myelopathy, infection, and tumor, and they remain indispensable for these indications [4,5]. However, a substantial proportion of contemporary neck pain presents not as a single discrete lesion, but as fluctuating, exposure-dependent dysfunction. Recent overviews of diagnostic reviews indicate that strong and consistent diagnostic indicators remain limited across many neck pain presentations, particularly in non-specific conditions [4,6]. This creates a practical mismatch between what current diagnostic tools are optimized to detect and what many patients experience during prolonged screen-based work, sustained postures, or repetitive low-load activity [2,3,4,6].

Routine radiography, computed tomography, and magnetic resonance imaging are fundamentally snapshot-based modalities. They characterize anatomy, alignment, and neural compression at isolated time points under standardized clinical conditions, but they do not directly capture fatigue-related deterioration, cumulative loading, exposure-dependent aggravation, or recovery dynamics over the course of daily life [4,5]. This limitation is especially relevant in neck disorders associated with digitally mediated behaviors, in which symptoms may develop gradually, fluctuate with task duration, and intensify only after prolonged exposure rather than during a brief clinical encounter [2,3,4]. Longitudinal evidence further suggests that cervical MRI findings have limited and inconsistent ability to predict future neck pain, reinforcing that morphology alone is an incomplete surrogate for symptom trajectories in non-specific disorders [8].

The problem is compounded by persistent clinico-radiographic discordance. Degenerative imaging findings are common in asymptomatic individuals and become increasingly prevalent with age [7]. Conversely, symptomatic patients may demonstrate only modest structural abnormalities or findings that do not adequately explain functional deterioration, symptom volatility, or work-related aggravation [4,7,8]. Standard imaging therefore remains essential for excluding serious pathology, but is considerably less informative for characterizing the dynamic mechanisms through which symptoms emerge, fluctuate, and recover over time [4,5,8].

Motion-sensitive imaging partly narrows this gap, but does not eliminate it. Dynamic radiographic methods, including dynamic X-ray image processing, quantitative fluoroscopy, and dual fluoroscopic imaging systems, can quantify intervertebral motion with substantially greater anatomical specificity and temporal resolution than routine clinical examination [13,16,17]. Available repeatability data further suggest that, under standardized conditions, segmental cervical motion patterns can be measured reproducibly [18]. However, these methods remain episodic, infrastructure-intensive, and generally limited to brief scripted tasks. In radiographic applications, ionizing radiation further constrains their suitability for frequent or prolonged monitoring [13,16,17,18].

Emerging dynamic and load-sensitive MRI approaches provide additional evidence that posture- and motion-dependent abnormalities may be underestimated on conventional static imaging, particularly in selected conditions such as cervical myelopathy and instability-related presentations [29,30]. Dynamic MRI may reveal occult or extension-related cord compression and other functional changes not visible on neutral supine MRI alone, but current protocols remain heterogeneous, indications are still evolving, and access remains limited [29,30]. These approaches are therefore better understood as problem-solving tools for selected clinical questions than as scalable methods for continuous non-invasive cervical functional assessment in real-world settings.

Taken together, current cervical diagnostics are highly effective for identifying major structural disease, but they remain poorly aligned with time-dependent dysfunction. The unresolved need is therefore not simply better visualization, but a framework capable of linking reference-grade segmental cervical motion characterization with scalable longitudinal assessment under real-world conditions.

## 4. Segmental Cervical Motion as a Clinically Meaningful Functional Domain

Segmental cervical motion refers to the relative kinematics between adjacent vertebral levels, including their rotational and translational behavior during functional tasks. In contrast to global cervical range of motion, which summarizes head movement relative to the trunk, segmental cervical motion reflects how movement is distributed across the cervical spine and how individual levels contribute to the overall motion pattern [11,12,13]. Experimental and imaging studies indicate that the upper and lower cervical regions do not behave as mechanically equivalent units, but instead demonstrate distinct motion characteristics depending on the task and plane of movement [11,12]. These observations are important because they imply that similar global motion amplitudes may arise from substantially different internal segmental strategies [11,12].

This distinction has direct clinical relevance. Conventional range-of-motion assessment is useful for describing overall movement capacity, yet it provides limited information about how motion is shared between intervertebral levels [12]. Video-fluoroscopic work has shown that the global end-ranges of neck flexion and extension do not necessarily represent the maximum rotational ranges achieved by individual cervical joints, meaning that endpoint-based clinical assessment can obscure substantial intervertebral variability [12]. In addition, dynamic motion studies suggest that healthy cervical joints do not move in a simple or uniform manner, but instead exhibit distributed and time-varying patterns across the movement excursion [11,12]. From a translational perspective, this means that two individuals with similar global cervical range may nonetheless differ considerably in segmental contribution, coordination, and mechanical redistribution [11,12].

Segmental cervical behavior is also clinically meaningful because neck disorders frequently involve altered sensorimotor control rather than structural abnormality alone. Reviews of neck disorders have documented disturbances in cervical proprioception, head–eye movement control, and postural stability, while systematic review evidence indicates that joint position sense error is often elevated in people with neck pain compared with healthy controls [9,10]. These findings suggest that the cervical spine functions not only as a load-bearing structure but also as a sensorimotor interface whose altered performance may influence symptoms, movement adaptation, and rehabilitation response [9,10]. A segment-level perspective is therefore relevant because internal motion redistribution may represent one biomechanical substrate through which altered afferent input and motor-control disturbances are expressed clinically.

Emerging imaging evidence further supports the view that segmental biomechanics vary systematically with biological and pathological context. Dynamic fluoroscopic and related imaging studies have reported that disc degeneration is associated with altered continuous intervertebral motion patterns, while broader reviews of cervical kinematics under healthy and degenerative conditions indicate that motion characteristics differ across populations and disease states [14,31]. In parallel, dynamic fluoroscopy research in aging populations suggests that older asymptomatic adults may lose the consistent sequence of segmental contributions observed in younger individuals, further reinforcing that motion quality may contain clinically relevant information beyond static morphology alone [15]. Taken together, these findings support the view that segmental cervical motion is not merely a higher-resolution version of range-of-motion testing, but a distinct functional domain that may capture load sharing, compensation, and motion organization in ways that global measures cannot [12,14,15,31].

At the same time, segmental cervical motion is not directly observable in scalable real-world monitoring. Reference methods such as dual fluoroscopic imaging systems can quantify individual cervical-level motion with high anatomical specificity, but they remain episodic, infrastructure-intensive, and unsuitable for continuous daily-life use [13]. Conversely, wearable inertial sensing, motion capture, and video-based approaches are more scalable, yet they remain indirect and require segment-informed interpretation [19,20,21,22,23,24]. Accordingly, segmental cervical behavior in real-world applications should be treated not as a directly measured quantity, but as a latent segment-informed functional construct inferred from external signals, contextual information, and sparse reference-grade imaging anchors. This framing shifts the target away from continuous anatomical reconstruction toward robust estimation of motion patterns that reflect segmental contribution, coordination, drift, and recovery under real-world exposure conditions [12,13,14].

## 5. Existing Assessment Modalities: What They Measure and What They Miss

No currently available modality provides a complete solution for cervical functional assessment that simultaneously combines segment-level functional specificity, feasibility for longitudinal real-world monitoring, and low-burden deployment in everyday settings. Existing modalities differ substantially in their primary outputs, the conditions under which they are applied, and their ability to capture clinically meaningful behavior over time. Some are optimized for structural diagnosis or reference-grade segmental cervical motion characterization under controlled conditions, whereas others are better suited to repeated assessment of global posture or external head–neck kinematics in ecologically valid settings. This trade-off lies at the core of the translational problem addressed in this review [4,13,14,16,17].

To compare these modalities in a structured manner, Table 1 summarizes their primary outputs, segment-level functional specificity, suitability for longitudinal real-world monitoring, burden/accessibility, and main translational limitations.

**Figure 1:**
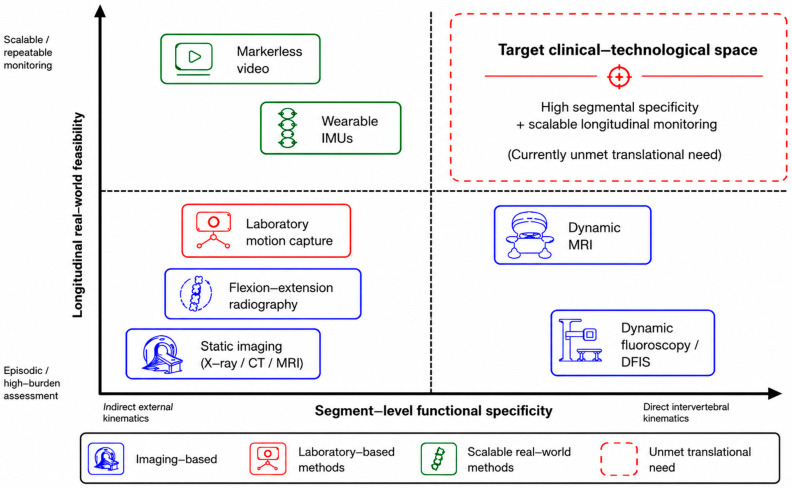
Trade-off between segment-level functional specificity and longitudinal real-world feasibility in cervical assessment.

While Table 1 provides a structured comparison of modality characteristics, Figure 1 visualizes the same landscape conceptually by positioning modalities along two translational dimensions: segment-level functional specificity and longitudinal real-world feasibility.

Existing cervical assessment modalities occupy different positions along a trade-off between segment-level functional specificity and longitudinal real-world feasibility. Imaging-based methods provide structural or segment-level precision but remain episodic and poorly suited to continuous monitoring in daily life. Laboratory motion capture enables controlled measurement of external head–neck kinematics but has limited ecological validity. Wearable inertial sensors and markerless video are more compatible with scalable real-world monitoring but remain indirect. The upper-right region represents the target clinical–technological space, where high segment-level functional specificity would be combined with scalable longitudinal real-world monitoring; this remains a currently unmet translational need.

Taken together, Table 1 and Figure 1 show that the modalities with the greatest segment-level functional specificity remain episodic and resource-intensive, whereas the modalities most compatible with scalable real-world monitoring remain indirect. The following subsections examine this trade-off in more detail, beginning with imaging-based modalities and then turning to laboratory and scalable external-sensing approaches.

### 5.1. Static Imaging and Functional Radiographs

At the clinically indispensable but functionally limited end of the imaging spectrum, static radiography, computed tomography, and magnetic resonance imaging remain central to cervical care, particularly for identifying fracture, deformity, degenerative narrowing, neural compression, myelopathy-related structural compromise, infection, or tumor [4,5]. Their principal strength lies in anatomical definition and diagnostic safety, especially when serious structural pathology must be ruled out. However, these modalities primarily characterize morphology and alignment rather than time-dependent cervical functional behavior [4,5,8]. This becomes a major limitation when symptom trajectories depend on exposure duration, repeated low-load activity, cumulative fatigue, or context-specific aggravation rather than on a single discrete lesion [2,3,4,8].

Flexion–extension radiographs partly extend static imaging by introducing task-dependent positional change, but their functional value remains limited. In most cases, they provide only sparse end-range snapshots under constrained testing conditions and cannot resolve the continuous cervical intervertebral motion patterns that may emerge during daily-life activity [12,32]. They are therefore better understood as adjunctive tools for selected instability-related questions than as methods for longitudinal functional phenotyping [32].

### 5.2. Dynamic Radiographic Imaging as a Reference for Segmental Cervical Motion Characterization

Among currently available methods, dynamic radiographic methods provide the strongest basis for reference-grade segmental cervical motion characterization. Methods such as dynamic X-ray image processing, quantitative fluoroscopy, and dual fluoroscopic imaging systems enable measurement of intervertebral kinematics with substantially greater anatomical specificity and temporal resolution than routine examination or static imaging [13,16,17]. These methods have been used to characterize segmental cervical motion under healthy and pathological conditions, to evaluate continuous cervical intervertebral motion patterns, and to examine repeatability under standardized experimental tasks [13,15,18,31].

Their main translational value lies in revealing motion distribution and intervertebral kinematics that are inaccessible to ordinary clinical testing. For this reason, dynamic radiographic imaging is relevant not only for mechanistic studies but also for anchoring and analytically evaluating candidate digital measures derived from external sensing. At the same time, its limitations are equally clear: imaging sessions are episodic, infrastructure-intensive, and generally restricted to brief scripted tasks. In radiographic approaches, ionizing radiation further constrains repeated use, particularly in longitudinal or ambulatory monitoring scenarios [13,16,17,18].

Dynamic or load-sensitive MRI contributes complementary posture- or motion-sensitive information in selected indications, including situations in which conventional neutral imaging may underestimate clinically relevant abnormalities [29,30]. However, it should be understood primarily as a functional problem-solving modality rather than as a direct reference for intervertebral kinematics. Even when dynamic or load-sensitive MRI reveals posture- or motion-dependent abnormalities, it remains not scalable for continuous real-world observation [29,30]. Taken together, dynamic radiographic methods are best regarded as reference approaches for targeted segmental cervical motion characterization and validation, whereas dynamic or load-sensitive MRI remains a complementary problem-solving modality. Neither approach provides a practical backbone for continuous real-world monitoring.

### 5.3. Laboratory Motion Capture, Wearable Sensors, and Markerless Video

Laboratory-based optical motion capture systems provide high-quality measurements of global external head–neck kinematics and remain valuable for biomechanical research under controlled conditions [19]. However, they provide only indirect information about cervical functional behavior from external markers and are constrained by laboratory setup, limited ecological validity, and the practical burden of controlled testing. Their ability to characterize real-world cervical function over extended periods is therefore limited, even when measurement precision is high [19].

Wearable inertial measurement units (IMUs) and related sensor platforms offer a different set of advantages. They are relatively scalable, increasingly affordable, and suitable for repeated or continuous data collection outside laboratory settings. Recent studies support their validity and reliability for cervical range-of-motion assessment and related head–neck kinematic measurements, including validation against optical reference approaches and the availability of openly described multiplanar motion datasets [20,21,22,33]. From a translational perspective, wearables are particularly attractive because they enable monitoring during daily routines, exposure windows, and repeated standardized tasks. Yet this scalability comes with an important limitation: they measure external head–neck kinematics rather than vertebral-level motion itself. Soft-tissue displacement, sensor-placement variability, and the underdetermined relationship between external signals and internal segmental cervical behavior make direct segment-level interpretation fundamentally challenging [20,21,22,33].

Markerless video-based motion analysis and pose-estimation methods further improve scalability by reducing hardware burden and enabling remote or semi-remote assessment [23,24]. These approaches may be particularly relevant for telemedicine, screening, and home-based rehabilitation contexts. However, they are sensitive to lighting conditions, camera placement, occlusion, clothing, context variability, and privacy considerations, and, like wearables, they generally capture global posture and external head–neck kinematics rather than direct intervertebral kinematics [23,24]. Thus, although both wearable and video-based approaches are promising for real-world monitoring, they require cautious segment-informed interpretation.

### 5.4. Translational Implication

Taken together, existing modalities are complementary rather than interchangeable. Static imaging remains essential for structural diagnosis and clinical safety; dynamic radiographic imaging provides reference-grade segmental cervical motion characterization; and laboratory, wearable, and video-based approaches enable progressively more scalable assessment of external head–neck kinematics and posture. This asymmetry defines the central translational gap: reference-grade methods provide segment-level functional specificity, whereas scalable methods provide longitudinal real-world feasibility. This asymmetry motivates the framework developed in the next section [13,16,17,19,20,21,22,23,24].

## 6. A Four-Layer Framework for Continuous Non-Invasive Cervical Functional Assessment

**Figure 2:**
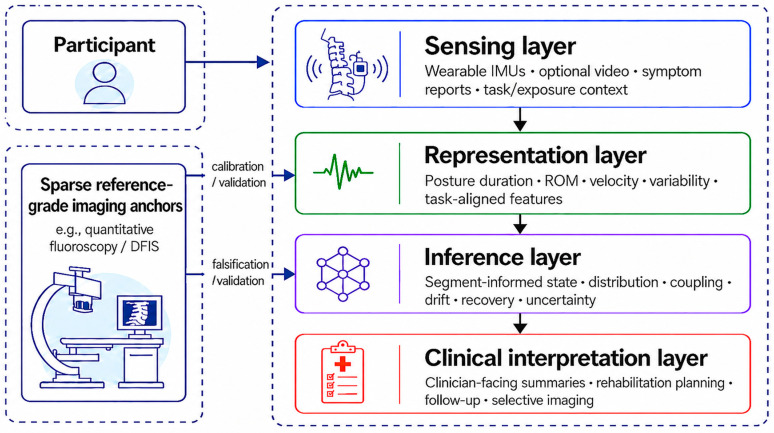
Data-flow structure of the four-layer framework for continuous non-invasive cervical functional assessment.

Building on the modality asymmetry outlined above, we propose a four-layer framework for continuous non-invasive cervical functional assessment structured as sensing → representation → inference → clinical interpretation [34,35,36]. The framework treats segmental cervical behavior as a clinically meaningful latent segment-informed functional construct and focuses on interpretable motion-related outputs that summarize segmental contribution, coordination, drift, and recovery under real-world conditions. As illustrated in Figure 2, scalable external sensing is transformed into structured representations and uncertainty-aware inference, while sparse reference-grade imaging anchors support calibration, validation, and falsification. Participant-level outputs should therefore be interpreted together with task context, symptom history, and data-quality constraints.

The figure illustrates how scalable inputs, including wearable inertial sensors, optional video, symptom reports, and task or exposure context, pass through the four framework layers. These inputs are first collected in real-world settings and then transformed into structured descriptors such as posture-duration profiles, range of motion (ROM), movement variability, and task-aligned features. The inference layer maps these descriptors to latent segment-informed functional states, such as regional motion distribution, coupling patterns, fatigue-related drift, recovery dynamics, and uncertainty estimates. The clinical interpretation layer translates these outputs into clinician-facing summaries that may support rehabilitation planning, ergonomic modification, follow-up timing, or selective imaging decisions when clinically justified. Sparse reference-grade imaging anchors, such as quantitative fluoroscopy or dual fluoroscopic imaging systems (DFIS), support calibration, analytical validation, falsification, and uncertainty assessment rather than continuous monitoring.

### 6.1. Sensing Layer: Real-World Data Acquisition

The sensing layer focuses on signals that can be collected repeatedly or continuously in real-world settings with acceptable burden, adherence, and feasibility. Candidate inputs include wearable inertial measurement units distributed across the head–neck–upper thorax region, optional video-based tracking, smartphone-derived data on task or exposure conditions, patient-reported symptom or fatigue logs, and, where appropriate, adjunct physiological or neuromuscular measures such as heart rate variability or surface electromyography [20,21,22,23,24,33,34]. The role of this layer is to capture scalable external, contextual, and optional physiological signals relevant to cervical functional assessment.

A key design principle at this stage is context of use. Sensor selection should be driven by the intended application, such as monitoring during prolonged screen-based work, rehabilitation follow-up, early symptom fluctuation detection, or telemedicine-supported home assessment [34,35,36]. Signal richness must therefore be balanced against burden, privacy, usability, battery requirements, and deployment constraints. More sensors do not necessarily yield greater translational value if adherence declines or if the resulting system becomes impractical outside research settings.

#### 6.1.1. Use Cases and a Minimal Standardized Task Battery

To avoid a decontextualized interpretation of cervical functional assessment, data collection should be framed around explicit use cases. Two especially relevant scenarios are fatigue-sensitive assessment during digitally mediated near-work and longitudinal monitoring of recovery trajectories during rehabilitation or post-intervention follow-up. Within these contexts, passive or semi-passive monitoring can be complemented by a brief standardized task battery designed to probe cervical control under comparable conditions. Such a battery may include neutral posture holds, slow flexion–extension, axial rotation, and lateral bending tasks, interpreted together with recent exposure history and symptom response [20,21,22,33]. This approach allows longitudinal changes to be evaluated not only in terms of daily-life fluctuation, but also against repeatable functional probes that improve within-person comparability over time.

#### 6.1.2. Illustrative Clinical Scenario

An illustrative early clinical scenario would involve an adult office worker with chronic fluctuating non-specific neck pain related to prolonged computer and smartphone use. After routine clinical evaluation, no red-flag symptoms or structural pathology requiring urgent intervention are identified. The patient reports mild symptoms in the morning, but progressive neck discomfort, fatigue, reduced concentration, and reduced work tolerance after several hours of screen-based work. In this use case, the clinical question is not whether a single structural lesion is present, but whether daily exposure, posture, task persistence, and recovery patterns are associated with reproducible changes in cervical functional behavior.

Monitoring could be performed over a two-week period using a lightweight chain or array of inertial sensors distributed along the head–neck–upper thorax region to capture external head–neck kinematics and posture changes during selected work periods and during the brief standardized task battery described above. Additional contextual data could include self-reported pain intensity, perceived fatigue, duration of screen exposure, work breaks, sleep quality, ergonomic conditions, and recovery time after symptom aggravation. These data would be used not for continuous anatomical reconstruction, but as external head–neck kinematic and contextual signals from which structured descriptors can be derived.

For the clinician, the practical value would be to determine whether symptom aggravation is accompanied by prolonged static posture, reduced movement variability, fatigue-related drift, delayed recovery after work, or unstable repeated-task performance. This information could help distinguish whether poor recovery is more likely related to insufficient ergonomic adaptation, excessive exposure duration, inconsistent rehabilitation exercise performance, or a need to adjust the treatment and follow-up plan. The resulting outputs could support individualized rehabilitation planning, ergonomic modification, patient education, follow-up timing, and selective imaging decisions when clinically justified.

### 6.2. Representation Layer: From Raw Signals to Structured Descriptors

Raw multimodal sensor streams are not, by themselves, clinically meaningful. The representation layer therefore transforms external head–neck kinematic and contextual data into structured descriptors that are more robust to device-specific variability, missingness, and real-world noise. Examples include posture-duration profiles, movement variability indices, movement-cycle descriptors, symmetry measures, exposure-linked summaries, and task-aligned features derived from repeated standardized maneuvers [20,22,34,35].

This layer is particularly important because cervical functional assessment in daily-life settings depends on indirect and context-sensitive external signals. External sensors are affected by soft-tissue artifact, sensor displacement, environmental variability, and partially standardized task execution and behavior. The goal is therefore not to preserve all raw signal complexity, but to derive structured descriptors that are sufficiently stable, interpretable, and generalizable to support segment-informed inference. Contextual information, such as work episodes, screen exposure, symptom logging, time of day, or recovery intervals, should also be incorporated at this stage so that subsequent inference is conditioned on meaningful behavioral context rather than on isolated kinematic snapshots [34,35,36].

### 6.3. Inference Layer: Estimating Latent Segment-Informed Functional States

The inference layer maps structured descriptors to a latent segment-informed functional state across the cervical region. This may involve physics-informed models, probabilistic state-space approaches, multimodal sensor fusion, or machine-learning methods trained against reference data derived from sparse reference-grade imaging anchors [34,35,36]. Regardless of the modeling strategy, the central principle remains the same: segmental cervical behavior should be inferred probabilistically rather than treated as a directly observed quantity in ambulatory monitoring.

This distinction is essential because the inverse problem is fundamentally underdetermined. Different internal segmental strategies may produce similar external head–neck kinematics, while sensor-placement variability and soft-tissue artifact further limit direct interpretability [21,22]. Inference should therefore be framed probabilistically, with explicit uncertainty estimates, rather than as deterministic reconstruction of vertebral-level motion. In practice, the most clinically useful outputs are likely to be low-dimensional functional summaries rather than full vertebral-level motion trajectories.

Personalization is also likely to be central at this layer. Baseline motion organization, compensatory strategies, and exposure sensitivity may differ substantially between individuals. Accordingly, longitudinal within-person change detection may prove more meaningful than applying universal thresholds derived from cross-sectional population averages. This is consistent with broader thinking in digital measure development, where fit-for-purpose outputs are expected to be defined in relation to context, performance characteristics, and intended decision use rather than technological novelty alone [34,35,36].

### 6.4. Clinical Interpretation Layer: From Inference to Decision-Relevant Meaning

The final layer translates inferred latent states into outputs that are clinically interpretable and useful for longitudinal care. Clinicians do not require continuous streams of raw external head–neck kinematic data or opaque model states; instead, they require concise summaries that relate to symptom fluctuation, functional deterioration, recovery capacity, and treatment response. The role of this layer is therefore to convert segment-informed inference into decision-relevant descriptors that can support phenotyping, follow-up, and individualized management.

All outputs at this stage should be accompanied by explicit uncertainty, data-quality indicators, and interpretability limits. Factors such as missing data, atypical task execution, poor sensor adherence, or context mismatch should remain visible rather than hidden behind a single score. This is particularly important in cervical applications, where the proposed measures are intended to complement rather than replace clinical reasoning, history taking, physical examination, and imaging when indicated. In this sense, the four-layer framework provides a structured translational pathway for converting external sensing into clinically meaningful candidate digital measures [34,35,36].

Taken together, this framework provides a practical bridge between scalable real-world sensing and reference-grade segmental cervical motion characterization. By making uncertainty, data quality, interpretability, and clinical context explicit, it creates a basis for defining candidate digital measures for cervical assessment that are interpretable, falsifiable, and suitable for staged validation. The next section therefore turns from architectural principles to output definition, focusing on segmental motion signatures as candidate digital measures for longitudinal cervical assessment.

## 7. Segmental Motion Signatures as Candidate Digital Measures

The four-layer framework outlined above requires outputs that are clinically interpretable, technically measurable, and suitable for staged validation. Rather than continuous reconstructions of vertebral-level motion, the most useful targets are likely to be low-dimensional functional descriptors that summarize how cervical motion is distributed, organized, and modulated over time. We define these descriptors as segmental motion signatures: candidate digital measures derived from multimodal external sensing and intended to reflect clinically meaningful properties of segmental cervical behavior under specified tasks and contexts [12,17,34,35,36,37]. Box 1 summarizes their operational definition and examples of minimal viable outputs for early-stage studies.

Box 1 **Segmental motion signatures: operational definition and minimal viable outputs.**

**Segmental motion signature**: A time-indexed descriptor, or set of descriptors, derived from pose-related or externally inferred motion features that summarize clinically meaningful properties of segmental cervical behavior or regional cervical motion under specified tasks and contexts, with quantified uncertainty.

Examples of minimal viable outputs for early-stage studies:

1. **Segmental Distribution Index (SDI):** A summary descriptor quantifying how total motion is distributed across inferred cervical regions or segment-informed compartments during standardized tasks, with propagated uncertainty bounds. SDI may be useful as an early-stage research output because regional redistribution is more tractable to estimate and validate than exact per-level trajectory reconstruction. At this stage, SDI should be treated as a conceptual research construct rather than a clinically deployable metric.
2. **Fatigue Drift Slope:** The rate of change in SDI or coupling-related descriptors across a predefined exposure window, such as 30–60 min of near-work. This output is intended to capture whether motion organization remains stable during exposure or progressively redistributes in a fatigue-sensitive manner.
3. **Recovery Half-Time:** The time required for drift- or variability-related descriptors to return toward baseline following an exposure or intervention. This output is relevant because it operationalizes recovery dynamics rather than static capacity alone and may therefore be informative for resilience, work tolerance, and treatment response.
4. **Instability Proxy Score:** A composite descriptor reflecting task-to-task variability, reversals, and disruptions in inferred coupling patterns. This output should be interpreted with particular caution and evaluated only against sparse reference-grade imaging anchors or justified reference-grade segmental cervical motion characterization in targeted cohorts. It is proposed as a hypothesis-generating construct rather than a surrogate diagnosis of structural instability.

Importantly, these outputs are hypotheses to be tested, not established biomarkers or direct measurements of vertebral-level motion. All candidate outputs should be defined using pre-specified computation rules and versioned algorithms to ensure reproducibility and falsifiability

At this stage, the value of segmental motion signatures depends on explicit context of use, transparent computation, uncertainty quantification, and incremental clinical relevance [34,35,36,37]. The goal is therefore not to classify patients on the basis of inferred motion alone, but to generate structured longitudinal descriptors that can support phenotyping, monitoring, and hypothesis testing in cervical care.

A useful segmental motion signature should satisfy several criteria. It should be interpretable in biomechanical and clinical terms rather than merely statistically discriminative; robust to real-world noise, missing data, and moderate sensor-placement variability; suitable for within-person longitudinal comparison; and falsifiable against sparse reference-grade imaging anchors or reference-grade segmental cervical motion characterization, with explicit reporting of uncertainty. These requirements favor summary descriptors of segmental contribution, coupling, drift, and recovery over anatomically ambitious but weakly identifiable reconstructions of full vertebral-level motion trajectories [12,17,34,35,37].

### 7.1. Defining Segmental Motion Signatures

As summarized in Box 1, segmental motion signatures should be interpreted through three linked elements: external signal inference, task- or exposure-specific context, and quantified uncertainty. This context dependence is essential: the same individual may exhibit different motion organization during brief controlled maneuvers, prolonged near-work, recovery after exposure, or rehabilitation follow-up. Segmental signatures should therefore be interpreted relative to the behavioral conditions under which they are obtained rather than as fixed, decontextualized traits.

For operational clarity, the data required to derive such signatures can be grouped into three categories: motion signals, contextual information, and sparse reference-grade imaging anchors used during development or validation. Motion signals may include head, neck, and upper-thorax orientation, angular velocity, range of motion, movement variability, and timing features derived from wearable sensors or video-based tracking. Contextual information describes the conditions under which these signals are collected, such as a standardized task, prolonged screen-work exposure, a rehabilitation exercise, symptom aggravation, or a recovery period. Sparse reference-grade imaging anchors, when available, provide a way to evaluate whether the inferred descriptors are anatomically plausible and analytically defensible.

Higher-resolution reference data, when available, should be treated as validation targets rather than continuously available measurements in scalable monitoring settings. The practical calculation of segmental motion signatures should therefore focus on functional abstraction: reducing motion-related features and context into a limited set of clinically interpretable descriptors, such as regional motion distribution, coupling patterns, fatigue-related drift, recovery dynamics, or instability-like variability over time [34,35,37].

### 7.2. Minimal Viable Outputs for Early-Stage Studies

For early translational studies, minimal viable outputs should prioritize interpretability and validation feasibility over complexity. A practical starting point is a small set of candidate digital measures linked to plausible clinical questions, evaluated against sparse reference-grade imaging anchors, and refined iteratively as evidence accumulates. The purpose of these outputs is not to maximize descriptive richness, but to identify signal forms that are sufficiently stable, meaningful, and falsifiable to support early-stage evidence generation [34,35,36,37]. Their calculation should follow a pre-specified pipeline: signal quality control, alignment to standardized tasks or exposure windows, extraction of motion-related features, reduction into local, regional, or whole-cervical descriptors, and reporting with uncertainty and data-quality indicators.

### 7.3. Why Longitudinal Signatures May Be More Informative than Snapshot Measures

The proposed outputs differ from conventional snapshot measures because they are intended to capture change over time rather than capacity at a single clinical time point. This distinction is especially relevant in contemporary neck dysfunction, in which symptoms are often aggravated by cumulative exposure, task persistence, or impaired recovery rather than by an isolated loss of global range of motion [2,3,4,5,6,7]. A patient may therefore demonstrate seemingly normal global cervical range of motion in clinic while still exhibiting altered fatigue-related redistribution, delayed recovery, or unstable task-to-task control.

From a translational standpoint, the clinically informative question may not be simply how far the neck can move, but how inferred cervical motion organization changes during exposure and how it recovers afterward. This shift mirrors broader developments in digital health, where repeated real-world measures are increasingly valued for their ability to characterize variability, resilience, and response trajectories rather than only cross-sectional status [34,35,36,37]. In cervical applications, such a perspective may be especially relevant for fatigue-sensitive phenotypes, rehabilitation monitoring, and telemedicine-supported longitudinal care.

### 7.4. Interpretability, Falsifiability, and Reporting Requirements

Because segmental motion signatures are inferred rather than directly observed, their credibility depends on rigorous reporting. Candidate outputs should therefore be defined using pre-specified computation rules, stable algorithm versioning, and clear statements of uncertainty and data quality. They should not be reported as equivalent to validated vertebral-level kinematics. Instead, they should be framed as segment-informed functional measures whose biomechanical and clinical meaning is supported, but not guaranteed, by external sensing and sparse reference-grade segmental cervical motion characterization [12,17,34,35].

Falsifiability is equally important. A candidate signature should be regarded as non-informative if it cannot be reproduced across repeated standardized tasks, fails to show predefined analytical agreement with sparse reference-grade imaging anchors, or provides no clinically meaningful information beyond simpler global measures. This is not a weakness of the framework, but a necessary condition for scientific credibility. By defining candidate outputs conservatively and evaluating them stepwise, the field can avoid premature anatomical claims while still developing clinically relevant digital measures.

Taken together, segmental motion signatures provide a bridge between the four-layer framework and the validation strategy required for clinical translation. Their value depends on fit-for-purpose development, transparent reporting, and falsifiable evaluation. The next section therefore addresses how such measures should be evaluated within an explicit validation framework.

## 8. Validation Framework for Clinical Translation

For segmental motion signatures to become scientifically credible and clinically useful, they must be evaluated within an explicit validation framework rather than judged solely on technical novelty or apparent face validity. In the present context, the most appropriate methodological backbone combines three complementary perspectives: the BEST resource, which helps clarify whether a measure is being positioned as a biomarker, endpoint, or related clinical tool [38]; the V3 framework for verification, analytical validation, and clinical validation of sensor-based digital measures [35]; and the V3+ extension, which adds usability validation as a requirement for scalable and trustworthy deployment [36]. Within this framework, segmental motion signatures remain candidate digital measures whose clinical meaning must be established through staged validation.

**Figure 3:**
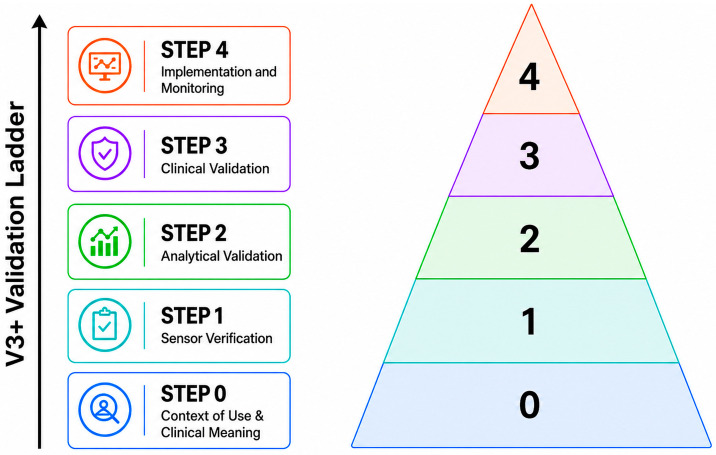
A V3+-aligned validation ladder for segmental motion signatures.

Figure 3 summarizes a stepwise validation ladder for clinical translation. The logic of this ladder is intentionally conservative: candidate outputs should first be shown to be technically reliable, then analytically defensible against reference-grade segmental cervical motion characterization, and only afterward evaluated for clinical meaning and real-world utility. This sequence is especially important in cervical applications, where the target of interest—segmental cervical behavior—is only indirectly observable in scalable ambulatory monitoring.

The proposed ladder progresses from context-of-use definition and sensor verification through analytical validation, clinical validation, and implementation monitoring. In this framework, Step 4 includes usability-focused implementation assessment, deployment readiness, and post-deployment monitoring. Transparent reporting, uncertainty quantification, and falsifiability apply across all stages.

### 8.1. Step 0: Context of Use and Clinical Meaningfulness

Validation should begin with an explicit definition of context of use. This includes the target population, intended application, expected task environment, comparator methods, and the clinical question to be informed. For cervical functional assessment, the most defensible early context of use is unlikely to be “all neck pain,” but a narrower setting such as fatigue-sensitive, fluctuating neck dysfunction in digitally exposed adults, particularly during prolonged screen-based work or structured rehabilitation follow-up [34,35,36,37]. Narrowing the initial context in this way improves interpretability, reduces construct drift, and makes validation targets more clinically coherent.

At this stage, it is also essential to pre-specify what would constitute clinically meaningful change. A measure that is technically stable but clinically uninterpretable has limited translational value. Candidate digital measures should therefore be prospectively linked to decision-relevant concepts such as symptom volatility, work tolerance, functional recovery, or response to rehabilitation. This does not require immediate proof of clinical benefit, but it does require that the measure be anchored to a plausible clinical role rather than presented as an abstract technical score [35,36,38].

### 8.2. Step 1: Verification

Verification addresses whether the sensing system and its components perform as intended from an engineering perspective [35]. In the present framework, this includes the hardware, firmware, synchronization logic, signal-processing steps, and software implementation used to derive segment-informed outputs. Relevant verification targets may include sampling stability, timing accuracy, signal drift, data loss, battery-related degradation, and tolerance to routine variation in sensor placement and use conditions.

For cervical applications, verification should also explicitly consider failure modes that are likely to influence downstream inference, including motion artifacts, detachment or displacement of sensors, task non-adherence, and inconsistencies in repeated task execution. Verification does not demonstrate clinical utility, but it establishes whether the data-generating system is technically reliable enough to support further validation.

### 8.3. Step 2: Analytical Validation

Analytical validation addresses whether the derived candidate digital measure accurately and reproducibly represents the intended construct relative to an appropriate reference [35]. For segmental motion signatures, this means evaluating agreement between inferred motion descriptors and reference-grade segmental cervical motion characterization using sparse reference-grade imaging anchors, such as quantitative fluoroscopy, dual fluoroscopic imaging systems, or other justified fit-for-purpose anchoring methods obtained under standardized conditions [13,16,17,18]. The goal is not to demand continuous ground truth for everyday monitoring, which is not feasible, but to determine whether the inferred outputs preserve clinically meaningful information with acceptable uncertainty.

Analytical validation should therefore focus on pre-specified outputs, such as the Segmental Distribution Index, Fatigue Drift Slope, Recovery Half-Time, or Instability Proxy Score, and test their performance across repeated tasks, exposure paradigms, and relevant hardware or deployment conditions. Important evaluation domains include repeatability, robustness to moderate sensor-placement variability, sensitivity to exposure-related change, and the extent to which estimated uncertainty is calibrated to observed disagreement with sparse reference-grade imaging anchors. At this stage, conservative analytical targets are preferable to anatomically ambitious claims that cannot be supported by the available evidence.

### 8.4. Step 3: Clinical Validation

Clinical validation evaluates whether a candidate digital measure is meaningfully associated with patient-relevant states or outcomes in the intended population and context of use [35]. For cervical functional assessment, this may include associations with pain intensity, disability, symptom fluctuation, work ability, or rehabilitation response. Importantly, clinical validation should not be limited to cross-sectional correlation. Because segmental motion signatures are explicitly longitudinal, stronger evidence will likely come from prospective studies showing that changes in segmental motion signatures track symptom trajectories, exposure tolerance, or recovery dynamics over time.

Clinical validation should also test incremental value. A candidate digital measure is more compelling if it explains meaningful variation beyond what is already captured by simple global range-of-motion measures, symptom self-report alone, or routine imaging findings. Conversely, if it adds no clinically relevant information beyond simpler or cheaper alternatives, its translational role may remain limited even if it is technically interesting [34,35,37]. This requirement is especially important in digital health, where technological complexity should not be mistaken for clinical usefulness.

### 8.5. Step 4: Usability Validation and Implementation Readiness

The V3+ framework emphasizes that a candidate digital measure is not truly fit for purpose unless it can be used reliably by intended users at scale [36]. In cervical functional assessment, this includes wearability, comfort, task adherence, burden of repeated measurement, clarity of instructions, compatibility with home or workplace use, and resilience to user diversity in body habitus, movement style, and digital literacy. A technically valid candidate measure that fails in routine use because of poor adherence, confusing workflows, or population-specific usability barriers is unlikely to succeed clinically.

Implementation assessment should also include monitoring for algorithm drift, systematic data-quality degradation, unexpected error patterns, and inequitable performance across subgroups or device ecosystems. These considerations are central to determining whether a candidate digital measure can move from proof of concept to trustworthy clinical support. In this sense, usability validation is not an optional extension of V3, but a necessary condition for real-world translation.

### 8.6. Candidate Success Criteria for Early Validation

For the validation ladder to be operational, success criteria should be defined prospectively within a specified context of use. Acceptable thresholds may differ across applications, such as rehabilitation follow-up, fatigue-sensitive monitoring during screen-based work, telemedicine-supported assessment, or post-intervention recovery tracking. At minimum, early validation should address technical reliability, analytical agreement with reference-grade segmental cervical motion characterization, clinical relevance, incremental value, and usability in the intended setting.

Table 2 summarizes candidate success criteria for early validation of segmental motion signatures.

A candidate segmental motion signature should therefore be considered reliable only when it is reproducible, analytically defensible, clinically meaningful within the intended use case, accompanied by calibrated uncertainty, and usable in the target setting.

### 8.7. Reporting Standards and Falsifiability

Validation must be accompanied by transparent reporting. If future implementations include an AI or machine-learning component that materially affects decision support, prediction, or index-test behavior, reporting of interventional studies should align with SPIRIT-AI for trial protocols and CONSORT-AI for trial reports [39,40]. Early-stage live clinical evaluations of AI-based decision-support systems may additionally benefit from DECIDE-AI, while diagnostic accuracy studies involving AI-centered index tests should align with STARD-AI [41,42]. These reporting frameworks do not replace V3 or V3+, but they improve transparency in study design, error analysis, applicability, and interpretation.

Falsifiability should also be stated explicitly. The success criteria described above should be accompanied by corresponding failure criteria, so that segmental motion signatures can be judged non-informative if they fail technical verification, fail to meet predefined analytical agreement criteria with sparse reference-grade imaging anchors, cannot be reproduced under repeated standardized conditions, or provide no clinically meaningful information beyond simpler measures. Such outcomes should be reported transparently rather than treated as negative obstacles to publication. A validation framework has value only if it allows the field to identify not only what works, but also what does not.

Taken together, this validation strategy reframes cervical digital measurement development as a staged evidence-generation problem rather than a purely technological one. By combining context-of-use clarity, V3/V3+ evaluation, transparent reporting, and explicit failure criteria, it supports the development of candidate digital measures that are interpretable, reproducible, and useful for longitudinal cervical functional assessment. The next section therefore considers how such measures may be integrated into telemedicine and responsible deployment pathways.

## 9. Telemedicine, Implementation, and Responsible Deployment

The translational value of segmental motion signatures lies not only in their analytical performance, but also in their potential to support longitudinal care outside conventional clinic-based assessment. Repeated non-invasive cervical functional monitoring may strengthen telemedicine by adding structured information beyond symptom self-report, enabling follow-up that is more sensitive to exposure-related aggravation, recovery dynamics, and treatment response over time [34,35,36,37].

At the same time, telemedicine use does not imply diagnostic automation. Segmental motion signatures should be understood as candidate digital measures that may enrich remote care pathways when interpreted within a broader clinical context. Their potential value lies in helping clinicians track deterioration during exposure, delayed recovery, or unstable repeated-task performance between visits, while complementing history taking, physical examination, conventional imaging when indicated, and clinician judgment.

### 9.1. Telemedicine Potential and Current Evidence

Current evidence suggests that telerehabilitation for neck pain is promising but not yet definitive. A recent Cochrane review reported low-certainty evidence that exercise-based telerehabilitation may slightly reduce neck pain and improve physical function compared with minimal intervention, while emphasizing the need for more high-quality trials. In parallel, a 2025 randomized controlled trial in young adult women with chronic neck pain and forward head posture found similar improvements in pain, neck disability, posture, and cervical range of motion with internet-based telerehabilitation and in-person supervised exercise, supporting feasibility in at least some targeted populations [43,44].

Within this context, segmental motion signatures should not be viewed as replacements for teleconsultation or rehabilitation, but as potential additions to remote monitoring and follow-up. Their greatest promise may lie in settings where clinicians need structured longitudinal information about fatigue-related deterioration, response to repetitive exposure, or pace of recovery between visits. When clinically contextualized and uncertainty-aware, such information may support triage, longitudinal phenotyping, and individualized adjustment of rehabilitation intensity.

### 9.2. Implementation in Real-World Workflows

For implementation to be clinically meaningful, data collection must fit naturally into real-world routines. Cervical functional monitoring systems requiring extensive setup, frequent recalibration, or high user burden are unlikely to scale outside research settings, regardless of technical sophistication. Practical deployment should therefore prioritize low-burden home or workplace monitoring, brief standardized probes where appropriate, passive or semi-passive acquisition when justified, low-friction user interfaces, and clinician-interpretable outputs [34,35,36,37].

In cervical applications, the most realistic early use cases are likely to be hybrid rather than fully autonomous: real-world acquisition combined with periodic clinician-in-the-loop review, symptom context, and selective use of sparse reference-grade imaging anchors when clinically justified. Such a model is more acceptable than high-burden systems that attempt exhaustive continuous anatomical measurement. Implementation success should therefore be judged by adherence, workflow compatibility, interpretability, and added value for routine clinical decision-making, not by technical performance alone.

Low-burden real-world acquisition supports derivation of segmental motion signatures that are reviewed within a clinician-in-the-loop workflow and translated into decision-support outputs rather than stand-alone diagnoses. Sparse imaging anchors in the pathway refer to sparse reference-grade imaging anchors used selectively for validation, calibration, or adjudication rather than as routine monitoring tools. Longitudinal adaptation links repeated monitoring to individualized follow-up. Responsible deployment depends on cross-cutting requirements of usability, privacy and governance, equity, transparency, and uncertainty-aware interpretation.

**Figure 4:**
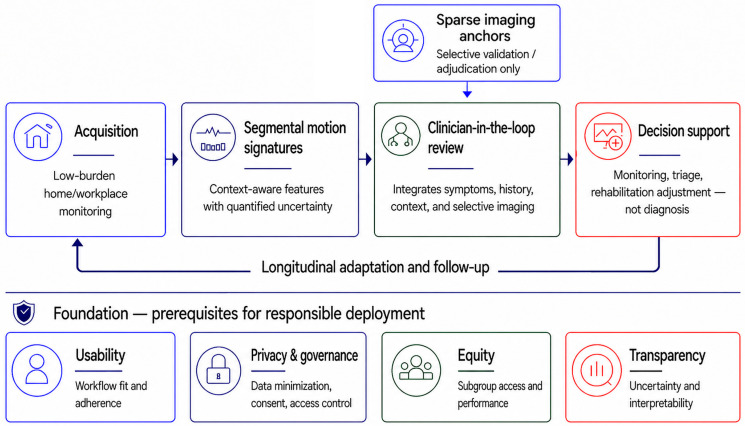
Translational pathway for responsible deployment of cervical digital measures in telemedicine.

Figure 4 therefore frames telemedicine deployment as a clinician-in-the-loop pathway in which longitudinal monitoring informs follow-up while preserving clinical context, selective imaging input, and uncertainty-aware interpretation.

### 9.3. Privacy, Data Governance, Equity, and Transparency

Responsible deployment requires explicit attention to privacy, governance, equitable access, and transparent communication of system limits. Continuous cervical functional monitoring may generate sensitive behavioral and health-related information, including posture habits, work rhythms, adherence patterns, and inferred health status. A recent scoping review of health-monitoring wearables identified recurring ethical and legal concerns spanning patient safety, autonomy, justice, and data protection. WHO guidance on artificial intelligence for health similarly emphasizes that ethics and human rights should remain central to the design, deployment, and use of digital systems in healthcare [45,46].

In practice, this means that candidate digital measures for cervical assessment should follow data-minimization principles, transparent consent processes, and role-appropriate access controls. Where feasible, feature extraction and quality checks should occur on-device or close to the point of collection, limiting unnecessary transmission of raw data. Patients should also understand what is being measured, what is being inferred, how uncertainty is handled, and which decisions are or are not supported by the system. Without this transparency, even technically valid monitoring may undermine trust or distort clinician–patient relationships.

Equity must also be treated as a core implementation requirement rather than a secondary consideration. Remote monitoring systems often assume stable internet access, compatible smartphones, sufficient digital literacy, and routine adherence capacity. However, recent evidence suggests that high device ownership does not necessarily translate into use of remote patient monitoring, medical apps, or wearables, particularly in underserved communities. Digital health technologies may therefore widen inequalities if implementation strategies do not explicitly address differential access, usability, adherence, and downstream benefit [47,48]. For cervical functional monitoring, this implies that implementation studies should evaluate not only average performance but also subgroup usability, adherence, interpretability, and failure modes across diverse users and settings.

### 9.4. Sparse Reference-Grade Imaging Anchors and Clinical Decision Support

Because the proposed framework relies on sparse reference-grade imaging anchors rather than continuous imaging, radiation stewardship remains essential. Dynamic radiographic methods may be justified for analytical validation, selective adjudication, or targeted clinical clarification, but they should be used only when the expected evidentiary or diagnostic gain outweighs the burden. In translational terms, the role of imaging is to support validation, calibration, falsification, or selective adjudication—not to become the routine monitoring backbone for real-world longitudinal care [13,16,17,18].

Consistent with the clinician-in-the-loop pathway shown in Figure 4, segmental motion signatures should remain decision-support measures rather than stand-alone diagnostic labels. Their purpose is to reduce the mismatch between fluctuating patient experience and snapshot-based assessment by adding longitudinal functional context to clinical reasoning. They should therefore complement history taking, physical examination, conventional imaging when indicated, and clinician judgment. This distinction is essential for avoiding over-interpretation: inferred deterioration without adequate context, uncertainty disclosure, or decision thresholds could increase false alerts, unnecessary escalation, or inappropriate reassurance. By contrast, a properly validated and transparently reported candidate digital measure may help clinicians identify patterns that are otherwise difficult to observe, such as fatigue-related drift, delayed recovery, or unstable repeated-task performance.

Taken together, telemedicine and remote monitoring create a meaningful translational opportunity for candidate digital measures in cervical assessment, but only if implementation is cautious, uncertainty-aware, and clinically contextualized. The evidence to date supports feasibility under selected conditions while also underscoring ethical obligations, equity challenges, and the need for clinician-interpretable outputs. These considerations frame the broader implications and limitations discussed in the next section.

## 10. Discussion

This review addresses a central mismatch in contemporary cervical assessment: many clinically relevant neck pain presentations are time-dependent, exposure-sensitive, and functionally variable, whereas dominant diagnostic pathways remain largely snapshot-based and structure-oriented [4,5,8]. The argument advanced here is not that existing cervical diagnostics are inadequate in general, but that they are incompletely aligned with disorders in which symptom expression depends on cumulative exposure, sustained posture, repetitive low-load activity, and recovery dynamics rather than on a single discrete structural lesion [2,3,4,8]. Within this context, segmental cervical motion emerges as a clinically meaningful but under-observed functional domain that may help narrow the gap between patient-reported symptoms and routine diagnostic readouts [11,12,13,14,15].

The principal contribution of this review is translational rather than purely descriptive. Rather than framing the problem as one of insufficient anatomical visualization alone, the review argues that a more realistic target for innovation is the development of clinically interpretable longitudinal measures derived from scalable external sensing and supported, where necessary, by sparse reference-grade imaging anchors [13,16,17,18,34,35,36,37,38]. This reframing shifts the field away from the unrealistic expectation that ambulatory monitoring should reproduce vertebral-level kinematics directly in daily life. Instead, it supports a more disciplined approach in which segmental cervical behavior is treated as a latent segment-informed functional construct that can be inferred with uncertainty, challenged analytically, and evaluated within explicit context-of-use constraints [34,35,36,37,38].

A clear distinction should therefore be made between evidence-supported premises and future-oriented proposals. Existing research supports several premises underlying this review: current cervical assessment remains limited for time-dependent and exposure-sensitive dysfunction; segmental cervical motion has biomechanical and clinical relevance; dynamic radiographic methods can characterize intervertebral motion with high anatomical specificity under controlled conditions; and wearable or video-based methods can provide scalable measurements of external head–neck kinematics. These elements provide the rationale for considering segmental cervical behavior as a clinically meaningful functional target. By contrast, segmental motion signatures remain proposed candidate digital measures whose clinical meaning, reproducibility, and incremental value must be established through staged validation.

This perspective also places cervical functional assessment within a broader movement in digital health. Increasingly, the most informative measures are not snapshot capacity metrics, but repeated descriptors that capture variability, deterioration under exposure, recovery, and treatment responsiveness over time [34,35,36,37]. For neck pain, this may be especially relevant because clinically important dysfunction often lies not only in how much motion is available at a single clinical time point, but in how motion is organized, redistributed, and recovered across repeated tasks and daily exposure conditions [2,3,4,11,12,13,14,15]. Segmental motion signatures may therefore prove useful not because they provide more data, but because they may provide data that are better matched to the time-dependent clinical profile of contemporary cervical disorders.

Several limitations should be emphasized. First, the proposed framework remains conceptual and translational rather than empirically established. Although the review integrates evidence from dynamic radiographic imaging, motion analysis, sensorimotor research, wearable sensing, validation science, and telemedicine, it does not present primary data validating any specific segmental motion signature for clinical use. Second, the inverse problem remains substantial. External head–neck kinematics do not uniquely determine internal segmental cervical behavior, and soft-tissue artifact, sensor placement, behavioral variability, and modeling assumptions may all influence inference [20,21,22,23,24]. Third, clinically useful outputs may ultimately prove more modest than initially envisioned. It is possible that coarse regional descriptors, rather than fine per-level reconstructions, will be the most robust outputs for real-world deployment. This should not be regarded as failure, but as an important boundary condition for responsible translation.

The narrative design of the review also warrants consideration. Although this design was selected to synthesize heterogeneous evidence across cervical biomechanics, dynamic radiographic imaging, scalable sensing, digital validation science, and telemedicine, it does not provide the methodological control of a systematic review or meta-analysis. No formal risk-of-bias assessment or quantitative evidence grading was performed; therefore, the synthesis may be affected by source-selection and interpretation bias. This limitation was partly mitigated by reporting the search domains, databases, source-selection principles, and approximate screening numbers, but future systematic or scoping reviews will be needed to evaluate specific subdomains in greater methodological depth.

An additional limitation concerns the heterogeneity and multifactorial nature of cervical disorders. Neck pain is not a single entity, and the value of segment-informed monitoring will likely differ across populations, phenotypes, and care settings. Measures that are informative in fatigue-sensitive, posture-related, or rehabilitation contexts may be less useful in acute trauma, dominant radiculopathy, or conditions in which structural diagnosis is already clear and management is not constrained by insufficient functional monitoring [4,5,35,36,38]. Cervical symptoms and disability should also not be reduced to motion quantification alone, because pain experience may be influenced by biological, biomechanical, neurological, psychological, behavioral, occupational, and social factors. Segmental motion signatures should therefore be interpreted as one component of broader clinical assessment, with a potential role in adding longitudinal functional context rather than replacing clinical reasoning. The field should resist the search for a universal cervical monitoring metric and instead prioritize fit-for-purpose development tied to specific clinical questions.

Despite these constraints, the framework proposed here has several practical strengths. It integrates currently fragmented literatures—cervical biomechanics, sensorimotor dysfunction, dynamic radiographic imaging, scalable sensing, digital validation science, and telemedicine—into a single translational structure [9,10,11,12,13,14,15,19,20,21,22,23,24,34,35,36,37,38]. It is also intentionally conservative: validation, uncertainty, usability, interpretability, and falsifiability are treated as central requirements rather than afterthoughts. This is important in a field where technical feasibility can easily be mistaken for clinical readiness. Useful candidate digital measures for cervical assessment will need to be not only measurable, but also interpretable, reproducible, decision-relevant, and deployable within ethically and practically acceptable workflows [35,36,37,38,45,46,47,48].

Several research priorities follow directly from this discussion. First, paired datasets are needed that link external multimodal sensing to reference-grade segmental cervical motion characterization using sparse reference-grade imaging anchors under clearly defined tasks and exposure paradigms. Second, candidate outputs such as motion distribution, drift, recovery, and variability measures should be evaluated prospectively in clinically relevant populations rather than only in technically convenient settings. Third, usability, adherence, subgroup performance, and uncertainty calibration should be assessed from the earliest stages of development rather than postponed until after technical prototyping. Fourth, longitudinal studies should test whether segmental motion signatures provide incremental value over simpler alternatives such as global range of motion, symptom diaries, or exposure history alone. Without such evidence, even technically elegant measures may remain translationally peripheral.

The implications for telemedicine are also notable. Remote care for neck pain is feasible in selected contexts, but current telemedicine pathways still rely heavily on self-report, intermittent observation, and broad functional assessment [43,44]. Segmental motion signatures could enrich these pathways by providing structured information about deterioration during exposure, delayed recovery, or unstable repeated-task performance between visits. However, any such deployment must remain grounded in privacy protection, equity-aware implementation, usability, and transparent clinician-in-the-loop decision support rather than automation for its own sake [45,46,47,48]. The purpose of candidate digital measures for cervical assessment should be to reduce diagnostic mismatch and improve longitudinal understanding, not to replace clinical judgment or create opaque over-monitoring.

Taken together, this review suggests that the future of cervical functional assessment may depend less on expanding anatomical visualization alone and more on inferring clinically meaningful longitudinal features from scalable sensing under rigorous validation constraints. If developed cautiously and evaluated transparently, segmental motion signatures may provide a practical bridge between real-world behavior and clinically meaningful motion characterization. Their ultimate value will depend not on conceptual appeal alone, but on whether they can demonstrate reproducibility, analytical defensibility, incremental clinical relevance, and real-world usability in the contexts for which they are intended.

## 11. Conclusions

Contemporary cervical assessment remains incompletely aligned with the time-dependent and context-sensitive nature of many modern neck disorders. Static imaging and brief in-clinic examination remain essential for structural diagnosis and clinical safety, but they are limited in their ability to characterize fatigue-related deterioration, exposure-dependent symptom fluctuation, and recovery trajectories over time [4,5,8]. At the same time, methods capable of reference-grade segmental cervical motion characterization are not suitable for scalable real-world monitoring, whereas wearable and video-based approaches offer real-world feasibility without direct access to vertebral-level kinematics [13,16,17,18,19,20,21,22,23,24].

This review argues that the most relevant translational target is therefore not continuous anatomical reconstruction, but the development of segmental motion signatures: candidate digital measures intended to summarize clinically meaningful properties of cervical motion distribution, coordination, drift, and recovery under specified tasks and contexts [12,17,34,35,36,37,38]. Framed in this way, segmental cervical behavior can be treated as a latent segment-informed functional construct inferred from multimodal external sensing and periodically anchored to sparse reference-grade imaging anchors.

Accordingly, the review provides three main contributions: a critical synthesis of current cervical assessment modalities and their translational limits; a four-layer framework for continuous non-invasive cervical functional assessment; and a validation-oriented pathway grounded in context of use, BEST terminology, V3/V3+ principles, and explicit falsifiability [35,36,38]. Together, these elements define a structured route from conceptual promise toward evidence-generating candidate digital measures for cervical assessment.

At present, segmental motion signatures should not be regarded as established biomarkers or stand-alone diagnostic entities. Their value depends on rigorous technical verification, analytical validation against sparse reference-grade imaging anchors, demonstration of incremental clinical relevance, usability in intended settings, and responsible deployment within longitudinal and telemedicine-supported care [35,36,38,43,44,45,46,47,48]. If developed cautiously and evaluated transparently, however, they may help reduce the persistent mismatch between fluctuating patient experience and snapshot-based assessment by enabling more context-sensitive, functionally grounded, and scalable monitoring of cervical dysfunction.

A Video Abstract is provided as Supplementary Materials to visually summarize the study rationale and proposed validation-oriented framework.

## Figures and Tables

**Table 1:** Comparison of cervical assessment modalities in terms of primary output, segment-level functional specificity, suitability for longitudinal real-world monitoring, burden/accessibility, and main translational limitation.

Modality	Primary Output	Segment-Level Functional Specificity	Longitudinal Real-World Monitoring	Burden/ Accessibility	Main Translational Limitation
X-ray/CT/MRI	Structural anatomy, alignment, neural compression	No direct segmental functional assessment	No	Variable; MRI often higher burden	Snapshot-based; limited linkage to time-dependent dysfunction
Flexion–extension radiographs	End-range positional change	Limited	No	Moderate	Sparse end-range information only; low temporal resolution
Dynamic radiographic methods/quantitative fluoroscopy/DFIS	Intervertebral kinematics	High; reference-grade	No	Limited access; radiation exposure	Scripted tasks only; unsuitable for scalable daily-life monitoring
Dynamic/load-sensitive MRI	Posture- or motion-sensitive structural change	Moderate to high in selected indications	No	Limited access; high burden	Heterogeneous protocols; episodic rather than continuous assessment
Laboratory motion capture	Global external kinematics	Indirect only	No	High	Controlled but low ecological validity; indirect segmental inference
Wearable IMUs	External head–neck kinematics	Indirect only	Yes	Low to moderate	Soft-tissue artifact; placement sensitivity; inverse problem
Markerless video	Global posture and external head–neck kinematics	Indirect only	Potentially	Low to moderate	Occlusion, lighting, privacy, and context variability

**Table 2:** Candidate success criteria for early validation of segmental motion signatures.

Validation Domain	Candidate Success Criteria
Verification	Data completeness, sampling stability, synchronization accuracy, limited drift, and sensor-placement tolerance
Analytical validation	Repeatability, agreement with sparse reference-grade imaging anchors, calibrated uncertainty, and robustness to moderate real-world variability
Clinical validation	Association with symptom fluctuation, functional limitation, exposure tolerance, work ability, or recovery dynamics
Incremental value	Added information beyond global range of motion, symptom self-report, exposure history, or routine imaging findings
Usability validation	Adherence, acceptable burden, clinician interpretability, and transparent data-quality and missing-data indicators

## Data Availability

The data supporting the results in this study are available on request from the corresponding author.

## Supplementary Materials

The following supporting information can be downloaded at: https://www.mdpi.com/article/10.3390/bioengineering13050584/s1, Video Abstract.

## Abbreviations

The following abbreviations are used in this manuscript:

AI	Artificial intelligence
BEST	Biomarkers, EndpointS, and other Tools
CONSORT-AI	Consolidated Standards of Reporting Trials–Artificial Intelligence
CT	Computed tomography
DECIDE-AI	Reporting guideline for the early-stage clinical evaluation of decision support systems driven by artificial intelligence
DFIS	Dual fluoroscopic imaging system(s)
IMU	Inertial measurement unit
MRI	Magnetic resonance imaging
PRISMA-ScR	Preferred Reporting Items for Systematic Reviews and Meta-Analyses Extension for Scoping Reviews
SANRA	Scale for the Assessment of Narrative Review Articles
SPIRIT-AI	Standard Protocol Items: Recommendations for Interventional Trials–Artificial Intelligence
STARD-AI	Standards for Reporting Diagnostic Accuracy Studies–Artificial Intelligence
V3	Verification, analytical validation, and clinical validation
V3+	Extension of the V3 framework including usability validation
WHO	World Health Organization
